# Thermal Stress Relaxation and High-Temperature Corrosion of Cr-Mo Steel Processed Using Multifunction Cavitation

**DOI:** 10.3390/ma11112291

**Published:** 2018-11-15

**Authors:** Masataka Ijiri, Norihiro Okada, Syouta Kanetou, Masato Yamamoto, Daisuke Nakagawa, Kumiko Tanaka, Toshihiko Yoshimura

**Affiliations:** Sanyo-Onoda City University, 1-1-1 Daigaku-Dori, Sanyo-Onoda, Yamaguchi 756-0884, Japan; f115018@ed.socu.ac.jp (N.O.); f115022@ed.socu.ac.jp (S.K.); f115019@ed.socu.ac.jp (M.Y.); nakagawa@rs.socu.ac.jp (D.N.); tanaka-k@rs.socu.ac.jp (K.T.); yoshimura-t@rs.socu.ac.jp (T.Y.)

**Keywords:** water jet peening, multifunction cavitation, Hot corrosion, Thermal stress cycle, Cr-Mo steel, embedding test, coating test

## Abstract

This research investigated high-temperature corrosion (500 °C) of Cr-Mo steel processed using water jet peening or multifunction cavitation (MFC), and the suitability of such steel for high-temperature boilers and reaction vessels. High-temperature corrosion was induced using an embedment test and a coating test using sulfide-type K_2_SO_4_-Na_2_SO_4_ powder. To measure the relaxation of the residual stress due to the decrease in work hardening caused by an increase in specimen temperature and the difference in thermal shrinkage between the surface and interior of the specimen, a thermal cycling test was conducted. For the MFC-processed specimen, the oxide film that formed on the surface suppressed mass loss, prevented crack formation, and reduced the compressive residual stress caused by high-temperature corrosion. MFC-processed Cr-Mo steel is thus suitable for a high-temperature corrosion environment.

## 1. Introduction

The importance of recycling has increased as waste disposal problems have become more serious. The concept of thermal cycle recycling has attracted attention as it allows the heat generated during incineration treatment to be recovered for power generation.

Highly efficient waste power generation is being actively promoted for the effective use of waste energy. However, in the superheater in a high-efficiency waste incineration boiler, molten salt containing chloride and sulfate forms in the ash attaches to the gas side pipe surface due to the high temperature of steam, causing high-temperature corrosion [1]. Further, when residual stress exists in a bent or welded portion of a pipe, high-temperature corrosion and local corrosion due to molten salt may be accelerated.

High-temperature and high-efficiency plants that utilize superheated steam (400 °C) have been constructed. In high-temperature boilers (400 °C or higher), the risk of corrosion damage increases significantly, and thus materials with high environmental resistance have been investigated. It is very important to manufacture materials with such resistance or apply a surface modification that provides it.

This present study focuses on water jet peening (WJP) technology, which utilizes cavitation. WJP is applied as a preventive maintenance technology in nuclear power plants [2,3]. WJP reduces the tensile residual stress in a structure, generated by welding or machining, to compressive residual stress. This prevents cracking due to stress corrosion and metal fatigue. However, it has been reported that the increase in pressure applied to Cr-Mo steel treated using WJP generates voids and cracks inside the specimen [4].

The present authors recently developed multifunction cavitation (MFC) [4,5,6,7] processing technology, which is a cavitation technique that applies ultrasonic waves to WJP. MFC can be used like WJP to reform a material surface. Improvements in residual stress, strength, and corrosion resistance have been reported for Ni-Cr-Mo steel [8], Cr-Mo steel [9,10], and Al alloy [11] processed using MFC. It has been shown that the corrosion resistance of the surface of Cr-Mo steel and the improvement in residual stress are affected by the period [12] and ultrasonic wave output [13] for specimens processed with MFC.

The present study investigates sulfide-based high-temperature corrosion (500 °C) of WJP- or MFC-treated Cr-Mo steel used for high-temperature boilers and reaction vessels.

## 2. Materials and Methods

### 2.1. Test Material and Processing Conditions

**Figure 1:**
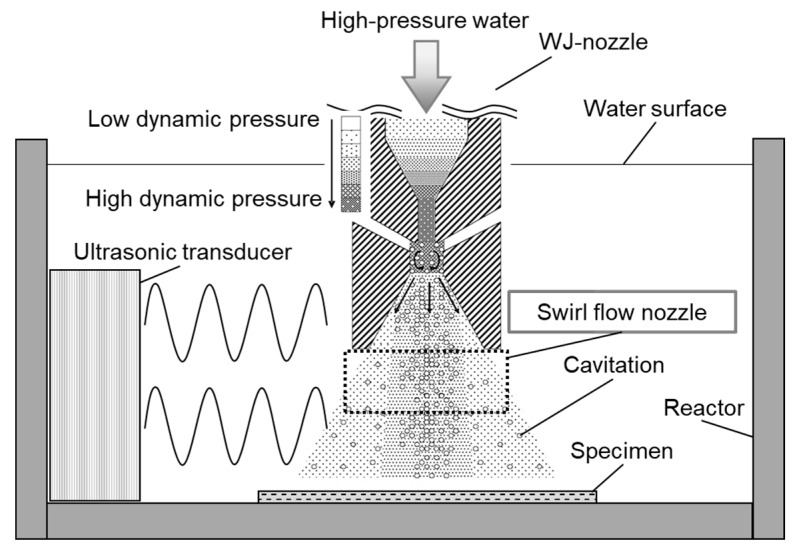
Equipment used for surface machining using water jet peening (WJP) with ultrasonic waves.

The material used for the tests was Cr-Mo steel, which is a structural machine steel. Its chemical composition is shown in Table 1. Rectangular specimens with dimensions of 100 × 100 × 3 mm^3^ were cut. Figure 1 shows the MFC processing equipment, in which an ultrasonic transducer (WD-1200-28T, Honda Electronics Co., Ltd., Aichi, Japan) emits acoustic pulses towards the water jet as it emerges from the nozzle. A swirl flow nozzle [14] is used at the tip of the WJP nozzle to increase the number and size of cavitation bubbles. The swirl flow nozzle [11,14] suppresses the erosion marks that form in the central part of a surface treated with WJP or MFC, and reduces surface damage due to cavitation bubbles. The discharge pressure of the pump was about 35 MPa, the nozzle diameter was 0.8 mm, and the distance between the nozzle and the specimen was assumed to be 65 mm. Processing was performed in a tank (JIS-SUS310S) with dimensions of 41 × 44 × 60 cm^3^. In a previous study [12], the effects of ultrasonic cycle conditions on the MFC-processed specimen surface were reported. It was found that dual mode most improved corrosion resistance and residual stress; dual mode was thus used in the present study. The output in this mode was 800 W and the frequency was varied from 25 to 27 kHz in steps of 10 Hz. The processing time was 2 min for all specimens.

### 2.2. High-Temperature Corrosion Conditions

**Figure 2:**
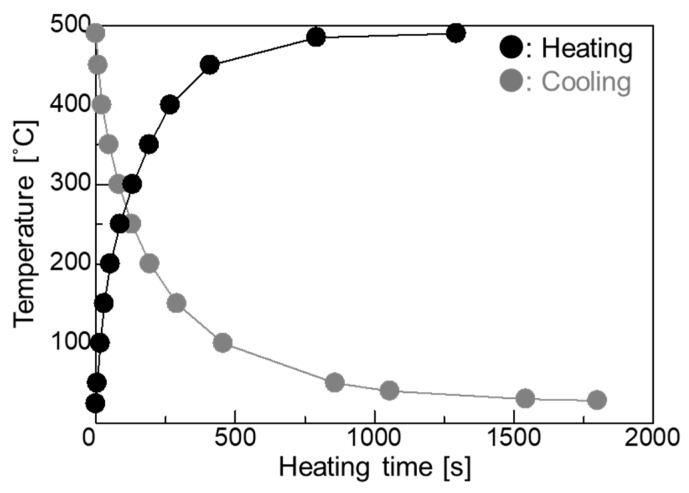
Relationship between heating time and temperature.

A thermocouple was welded to the specimen surface to determine the time at which the specimen surface temperature reached 500 °C. An electric furnace was heated to 500 °C, and the specimen was inserted until its temperature reached 500 °C. The relationship between the specimen surface temperature and the heat treatment time is shown in Figure 2. This condition was adopted as the condition for high-temperature corrosion since the temperature reached around 500 °C at about 7 min 50 s. To test corrosion, an embedding test and a coating test were adopted. A mixed ash of K_2_SO_4_-Na_2_SO_4_ at a weight ratio of 1:1 was used. The melting point of this synthetic ash was above 870 °C, as determined from the corresponding phase diagram [15]. Using the embedding test, Cr-Mo steel was placed at the bottom of an alumina crucible (height: 67 mm; outside diameter: 52 mm; capacity: 90 mL), covered with the synthetic ash, and heated in an electric furnace at 500 °C in the atmosphere. After heating, the specimen was immersed in an aqueous solution of sodium hydroxide (18%), potassium permanganate (3%), and pure water (79%) for 15 min to remove the oxide scale attached to the specimen surface. The specimen was then immersed in an aqueous solution of diammonium hydrogen citrate (10%) and pure water (90%) for 15 min. Finally, the specimen was cleaned with an ultrasonic washer. In the coating test, 5 g of mixed ash and 20 mL of acetone were placed in a beaker and stirred for 5 min with an ultrasonic washer. Then, about 1.26 g of the mixture was applied to the specimen surface with a brush. The oxide scale was removed in the same way as done in the embedding test. The coating test and embedding test were each repeated 20 times. After the corrosion test, specimens were evaluated using mass loss measurements, optical microscopy (OM), and scanning electron microscopy (SEM). For OM observation, each specimen was mirror-polished and then corroded with 5 vol% nital.

### 2.3. Heat Cycle Conditions

Two kinds of stress are generated by heat treatment. The first is the thermal stress caused by the difference in thermal shrinkage between the surface and interior of a specimen. The second is the transformation stress that occurs when deformation is caused by the martensitic transformation due to the temperature difference between the surface and interior of a specimen. In order to remove this transformation stress, the time required for the specimen surface to cool to room temperature in the atmosphere after heating was measured; it was found to be 25 min 43 s. Twenty heating and cooling cycles were performed. After each thermal stress cycle, residual stress measurement and OM observation of a specimen section were carried out. Residual stress was measured using an X-ray stress analyzer (MSF-3M, Rigaku Co., Ltd., Tokyo, Japan) using the peak top method after measurement of the strain between (211) lattice planes with the Cr Kα line generated at 30 kV and 10 mA. A region of 1 × 1 cm^2^ was measured. Tensile residual stress was taken to be positive and compressive residual stress was taken to be negative.

## 3. Results and Discussion

**Figure 3:**
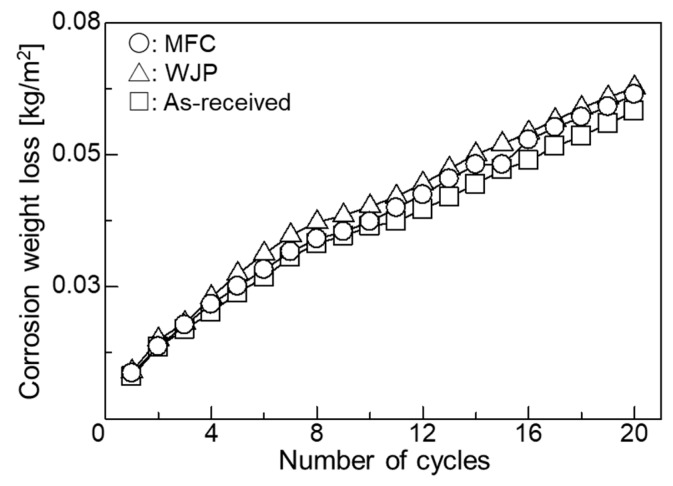
Relationship between corrosion weight loss and number of cycles in embedding test.

Figure 3 shows the relationship between corrosion loss and number of cycles in the embedding test. Corrosion weight loss was small; it was smallest for the as-received specimen, followed by the MFC-treated specimen and the WJP-treated specimen. OM observations (images not shown) indicated no change in phase transformation or particle size after heat treatment.

**Figure 4:**
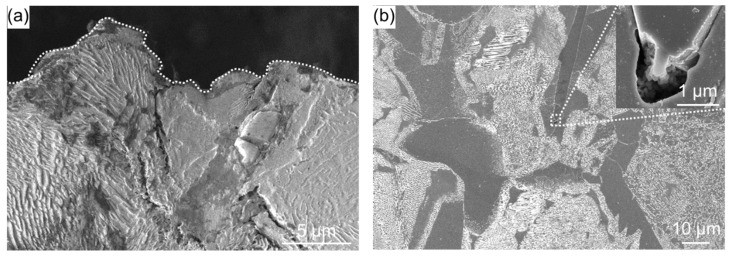
Cross-sectional SEM image of as-received specimen (**a**) near the surface (broken line is the specimen surface) and (**b**) away from the surface. Inset in (**b**) shows an enlarged SEM image of the region indicated by the dashed box.

Figure 4 shows cross-sectional SEM images of the as-received specimen after the embedding test. Figure 4a shows that several cracks formed on the surface. These cracks occurred at the grain boundaries. Cracks also formed near the surface. Figure 4b shows that voids formed at the grain boundaries of ferrite inside the specimen. In addition to the effects of high-temperature corrosion, the internal tension caused by the temperature difference between the surface and the interior generated during heating due to the difference in thermal shrinkage caused the stress corrosion cracking and voids. The cracks propagated mainly from grain boundaries. Grain boundary stress corrosion cracking may have occurred.

**Figure 5:**
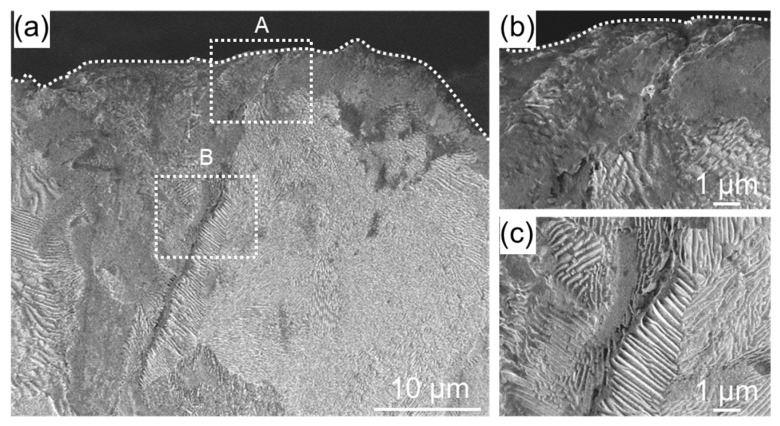
(**a**) Cross-sectional SEM images of a specimen after WJP treatment for 2 min. Enlarged SEM images of the regions indicated by dashed boxes (**b**) A and (**c**) B in (**a**). The dashed lines indicate the specimen surface.

Figure 5 shows cross-sectional SEM images of a WJP-processed specimen after the embedding test. Compared with Figure 4, the cracks are narrower and do not branch. The cracks formed along the grain boundaries of pearlite and ferrite. Grain boundary stress corrosion cracking may have occurred, as in the as-received specimen. The WJP-processed specimen had fewer cracks at the surface compared to the as-received specimen. Improvement in residual stress has been reported for WJP-treated steel [9,10]. This compressive residual stress seems to suppress the thermal stress due to high-temperature corrosion.

**Figure 6:**
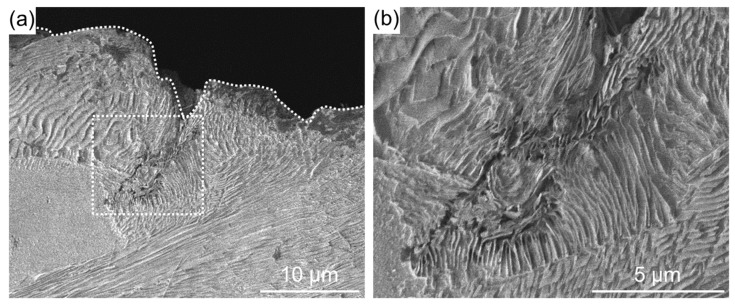
(**a**) Cross-sectional SEM image of a specimen after multifunction cavitation (MFC) treatment for 2 min; (**b**) Enlarged SEM image of the region indicated by the dashed box in (**b**). The dashed line indicates the specimen surface.

Figure 6 shows cross-sectional SEM images of an MFC-processed specimen after the embedding test. Although the width of the cracks is narrow, cracks on the surface did not propagate to the interior, unlike in Figure 5. A previous study [10] found that an oxide film forms on the surface when the surface potential of Cr-Mo steel is increased by MFC. Improvement in residual stress [9] has been reported for MFC-treated steel. The thermal stress due to high-temperature corrosion was alleviated by the heat-insulating effect of the coating and the compressive residual stress on the surface. It is assumed that the interior had no voids and cracks, and resisted the deformation caused by thermal stress.

**Figure 7:**
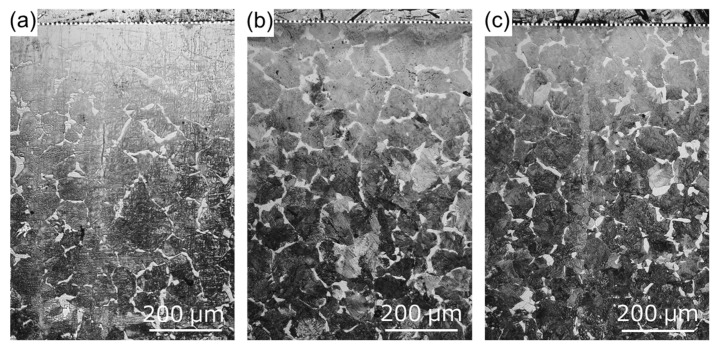
Cross-sectional OM images of (**a**) as-received specimen and specimens treated with (**b**) WJP and (**c**) MFC for 2 min after the thermal cycling test. The dashed lines indicate the surface of each specimen.

To measure the residual stress due to thermal stress, the as-received and treated specimens were thermally cycled in an electric furnace at 500 °C under the atmosphere. The residual stress measurement results are shown in Table 2. For the as-received specimen, tensile residual stress decreased after heat treatment. For the WJP- and MFC-processed specimens, compressive residual stress decreased after heat treatment; it decreased more for the WJP-processed specimen. In order to clarify the cause of the stress decrease, cross-sectional OM images of the specimens are shown in Figure 7. For all specimens, pearlite (black) changed to ferrite (white) near the surface. The OM observations were conducted under the same illumination intensity for all specimens. Diffusion decarburization likely occurred. The surface of the MFC-processed specimen had a thin decarburized layer. It has been reported that the improved corrosion resistance of an MFC-processed surface is due to the formation of an oxide film [10]. Since this oxide film suppresses heat transfer from the surface to the interior, it is considered that diffusion decarburization from the surface to the interior was less than that in other specimens. When a decarburized layer forms near the surface, since thermal expansion becomes larger than that for the normal part where thermal stress is low, the tensile stress increases and cracks are likely to form. However, for all specimens, no cracking occurred on the surface after thermal cycling. This is likely due to the low number of thermal cycles and the low temperature applied to the surface. If either is increased, cracks will eventually form on the surface. It has been reported that the high-temperature corrosion resistance improves when a dense oxide film exists on a heat-resistant alloy surface [16]. For the MFC-processed Cr-Mo steel [10], an oxide film formed on the surface, but iron oxide formed at a depth of 200 μm below the surface. In the embedding test, for the MFC-process specimen, the cross section was corroded instead of the surface, so its corrosion weight loss, shown in Figure 3, is slightly less than that for the as-received specimen. A high-temperature corrosion cycle was carried out in the coating test.

**Figure 8:**
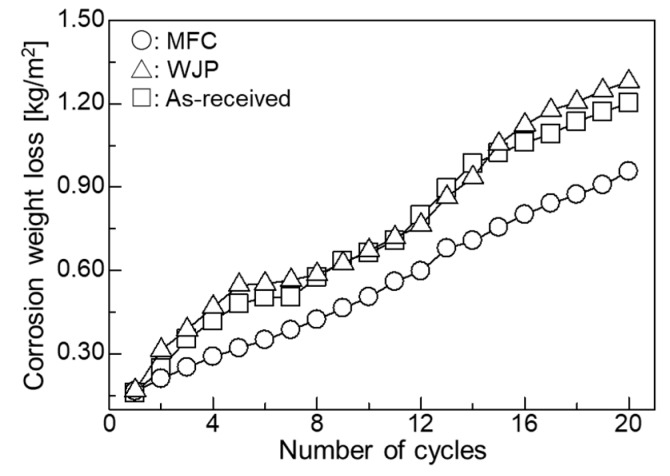
Relationship between corrosion weight loss and number of cycles in the coating test.

Figure 8 shows the relationship between the corrosion loss and the number of cycles in the coating test. The corrosion weight loss was small; the MFC-treated specimen had the smallest weight loss, followed by the WJP has larger weight loss than as-received. The results for high-temperature corrosion of the MFC-processed specimen for the coating test are different from those for the embedding test.

**Figure 9:**
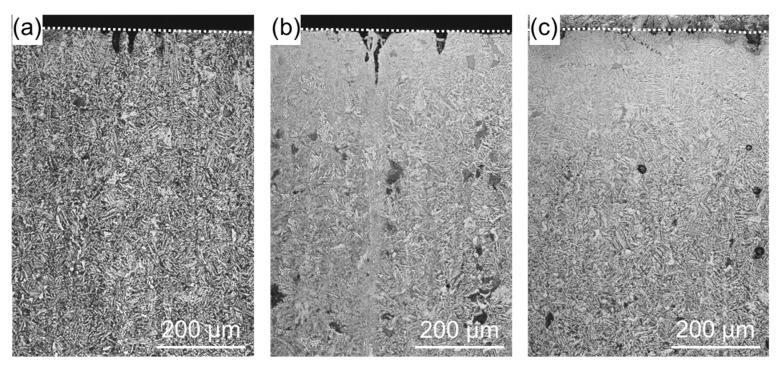
Cross-sectional OM images of (**a**) as-received specimen and specimens treated with (**b**) WJP and (**c**) MFC for 2 min after the coating test. The dashed lines indicate the surface of each specimen.

Figure 9 shows cross-sectional OM images of the specimens after the coating test. Unlike the as-received specimen, the treated specimens exhibited a microstructure mix of cementite and ferrite after high-temperature corrosion. Cementite may have transformed into spheroidized cementite when pearlite and ferrite were annealed at 500 °C; it eventually became spheroidized cementite if heating was conducted for a long period. In the embedding test, it took time for the whole container to warm up, and adhesion between the powder and the sample was poor; the mass reduction was thus small. On the other hand, in the coating test, there was no container for the powder and the specimen received heat directly in the electric furnace. It is thus considered that the surface cracked because the thermal stress increased and adhesion between the powder and the specimen was strong.

**Figure 10:**
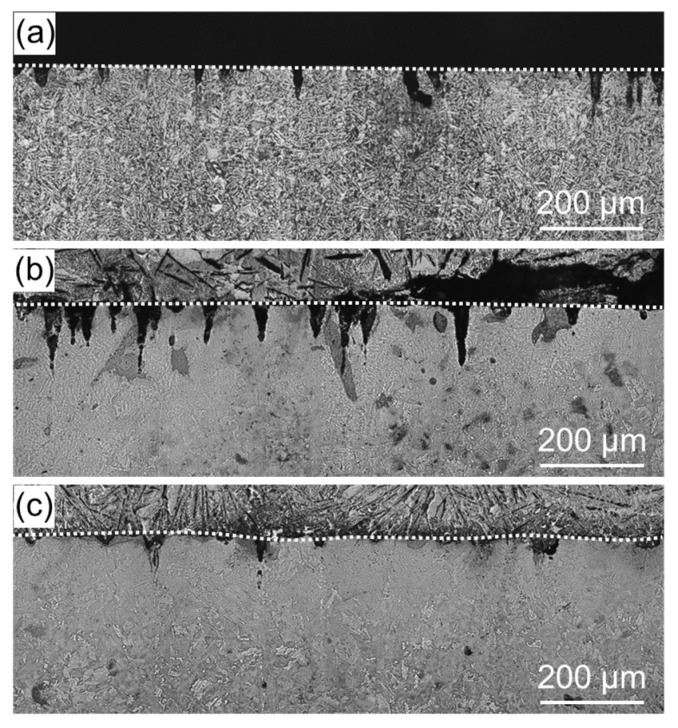
Enlarged cross-sectional OM images (**a**) as-received specimen and specimens treated with (**b**) WJP and (**c**) MFC for 2 min after the coating test. The dashed lines indicate the surface of each specimen.

Enlarged images of the vicinity of the specimen surface are shown in Figure 10. For the as-received specimen, large cracks did not occur in the vicinity of the surface, but the amount of high-temperature corrosion was large. For the WJP-processed specimen, the corrosion amount was large, as with the as-received specimen, and the density of surface cracks was the highest of all specimens. For the MFC-processed specimen, the amount of high-temperature corrosion was small and the density of surface cracks was low.

MFC-processed Cr-Mo steel is suitable for an environment with high-temperature corrosion (e.g., a sulfide system) for the following reasons.

- The oxide film that forms on the specimen surface reduces the compressive residual stress and decreased the heat transfer from the surface to the interior.
- Since voids and cracks are unlikely to form in the interior, cracks that can be generated by thermal stress caused by high-temperature corrosion are unlikely to occur.

The results show that MFC treatment suppresses the high-temperature corrosion (500 °C) of Cr-Mo steel in a sulfide system environment.

## 4. Conclusions

This study investigated sulfide-based high-temperature corrosion (500 °C) of WJP- and MFC-processed Cr-Mo steel used for boilers and reaction vessels. In the embedding test, there was almost no change in corrosion weight loss, but cracks formed in the as-received and processed specimens. The cracks in the as-received and WJP-processed specimens propagated from the surface to the interior, indicating grain boundary stress corrosion cracking. In the thermal cycling test, Residual stress decreased in the as-received and processed specimens. The MFC-processed specimen exhibited the least change in residual stress. Diffusion decarburization likely affected all specimen surfaces. Compared with the WJP-processed specimen, an oxide film more easily formed on the MFC-processed surface, so the thermal stress transmitted from the surface to the interior was low, and thus the change in residual stress was small. In the coating test, the corrosion loss was smallest for the MFC-treated specimen, followed by the as-received and WJP-treated specimens. This is related to the oxide film that formed on the surface. The MFC-processed specimen was found to have the highest high-temperature corrosion resistance.

## Figures and Tables

**Table 1:** Chemical composition of Cr-Mo steel (mass%).

C	Si	Mn	P	Ni	Cr	Mo	Cu	Fe
0.37	0.32	0.81	0.014	0.012	0.95	0.15	0.14	Bal.

**Table 2:** Residual stress in specimens after thermal cycling.

	After Machining (MPa)	After Thermal Cycling (MPa)
As received	+147.33	+43.14
WJP	−366.78	−79.83
MFC	−409.58	−272.90
